# Implementation and Outcomes of a Perioperative Geriatrics Strategy, PRIME, for Older Adults Undergoing Gastrointestinal Cancer Surgery

**DOI:** 10.3390/curroncol32090494

**Published:** 2025-09-03

**Authors:** Gabriella Jacob, Eric K. C. Wong, Rachel Fuh, Tyler R. Chesney, Camilla L. Wong

**Affiliations:** 1 Division of Geriatric Medicine, Department of Medicine, University of Toronto, 6 Queen’s Park Crescent West, Toronto, ON M5S 3H2, Canada; gabi.jacob@mail.utoronto.ca (G.J.);; 2 Li Ka Shing Knowledge Institute, St. Michael’s Hospital, 209 Victoria St, Toronto, ON M5B 1X3, Canada; tyler.chesney@unityhealth.to; 3 Division of Geriatric Medicine, St. Michael’s Hospital, 30 Bond Street, Toronto, ON M5B 1W8, Canada; 4 Faculty of Arts and Science, Queen’s University, 99 University Avenue, Kingston, ON K7L 3N6, Canada; 21ref8@queensu.ca; 5 Division of General Surgery, Department of Surgery, University of Toronto, 149 College Street, Toronto, ON M5T 1P5, Canada

**Keywords:** geriatric assessment, frailty, older adults, colorectal cancer, perioperative medicine, geriatric oncology

## Abstract

Introduction: The number of older adults living with frailty undergoing gastrointestinal cancer surgery is increasing. To address the unique needs of the population, a whole pathway perioperative geriatrics strategy—PRIME—was developed to integrate geriatric principles into surgical care. The objective of this study was to evaluate the implementation of PRIME using validated structural, process, and outcome quality indicators. Materials and Methods: This retrospective cohort study included 106 consecutive patients aged 70 years and older who underwent gastrointestinal surgery for cancer or pre-cancerous lesions at a single institution between 1 July 2020 and 5 October 2023. The whole pathway perioperative geriatrics strategy, PRIME, includes preoperative comprehensive geriatric assessment (CGA), collaborative care between surgery, geriatrics, and anesthesia, and post-operative co-management. Implementation was evaluated using validated structural, process, and outcome quality indicators. Results: Most structural indicators (five of eight) were implemented. In terms of process indicators, 96.2% (*n* = 102) received CGA prior to or within 24 h of admission. Adherence to screening was high: 97.2% for dementia, 96.2% for functional status, and 95.3% for frailty. The median number interventions resulting from CGA was 17 (IQR 14–20). Serious complication, delirium, and functional decline occurred in 19.8%, 27.1%, and 19.8%, respectively. Conclusions: Implementation of a perioperative geriatrics strategy for older adults undergoing gastrointestinal cancer/pre-cancer lesion surgery is feasible, with high adherence to structural and process quality indicators.

## Simple Summary

Older adults undergoing gastrointestinal surgery for cancer or pre-cancerous lesions received a whole pathway perioperative geriatrics strategy which included preoperative comprehensive geriatric assessment, collaborative care between surgery, geriatrics, and anesthesia, and post-operative co-management. There was good adherence to validated structural and process quality indicators. Outcome quality indicators demonstrated that this strategy compares favorably with other studies.

## 1. Introduction

In North America, approximately two-thirds of cancers are diagnosed in adults over age 75 [1]. Surgery is the primary treatment for many gastrointestinal cancers. Frailty is a clinically identifiable state of diminished physiologic reserve with increased vulnerability to a broad range of adverse health outcomes including postoperative complications, longer length of stay, and increased mortality [2,3]. The prevalence of frailty among older adults undergoing colorectal surgery is 25–46% and 29% in gastric cancer [4,5].

Perioperative care guidelines from the British Geriatrics Society, Italian Intersociety Consensus and American College of Surgeons/American Geriatrics Society recommend screening for frailty and associated geriatric syndromes in all older adults undergoing surgery [6,7,8,9]. The American Society of Colon and Rectal Surgeons guidelines include a strong recommendation that patients living with frailty may benefit from a multidisciplinary approach to perioperative care that includes a health care provider with geriatric expertise [10]. The British Geriatrics Society specifically recommends a comprehensive geriatric assessment (CGA) for individuals with frailty [6]. Comprehensive geriatric assessment is a “multidimensional, multidisciplinary process which identifies medical, social and functional needs, and the development of a co-ordinated care plan to meet those needs” [11]. In the postoperative setting, guideline recommendations include delirium screening, early mobilization, interventions to prevent functional decline and pressure injury prevention. However, uptake of these recommendations in routine perioperative care remains low [12].

A systematic review of 13 randomized trials examining CGA in the perioperative setting found that detailed reporting of the components of the CGA, duration of intervention, personnel, and level of adherence to intervention is often lacking [13]. This limits the ability of other centers to develop and implement perioperative services for older adults.

Consequently, there is impetus to describe and evaluate the performance of perioperative geriatric care models using expanded criteria including structural and process quality indicators. An expert consensus, developed using Delphi methodology, identified eight structural, seven process, and sixteen outcome quality indicators for evaluating geriatric co-management models [14]. Structural indicators are system-related factors that contribute to the ability to deliver the intervention (e.g., hospital staffing), while process indicators assess the delivery of interventions to the patient (e.g., percentage of patients who are screened for frailty). Outcome indicators are health-related patient events that may be affected by the interventions (e.g., incidence of delirium). Process evaluation of complex interventions, such as the CGA, standardizes the design and delivery of such services, ensuring fidelity to evidence-based models. This evaluation includes describing the number and type of actions and interventions resulting from CGA such as new diagnoses, medication changes, therapy interventions, and advanced care planning [15].

The objective of this study was to evaluate the performance of a perioperative geriatrics care model for older adults undergoing surgery for gastrointestinal cancer or pre-cancerous lesions, using expert consensus-based and validated structural, process, and outcome quality indicators.

## 2. Materials and Methods

### 2.1. Study Design and Participants

This was a retrospective cohort study of consecutive older adults, aged 70 years and older, who underwent surgery for gastrointestinal cancer and related pre-cancerous lesions (high grade dysplasia, tubulovillous adenomas, or colonic polyps) at St Michael’s Hospital (Toronto, ON, Canada) between 1 July 2020 and 5 October 2023. Elective and urgent, but not emergency, surgeries were included.

### 2.2. Intervention

**Figure 1:**
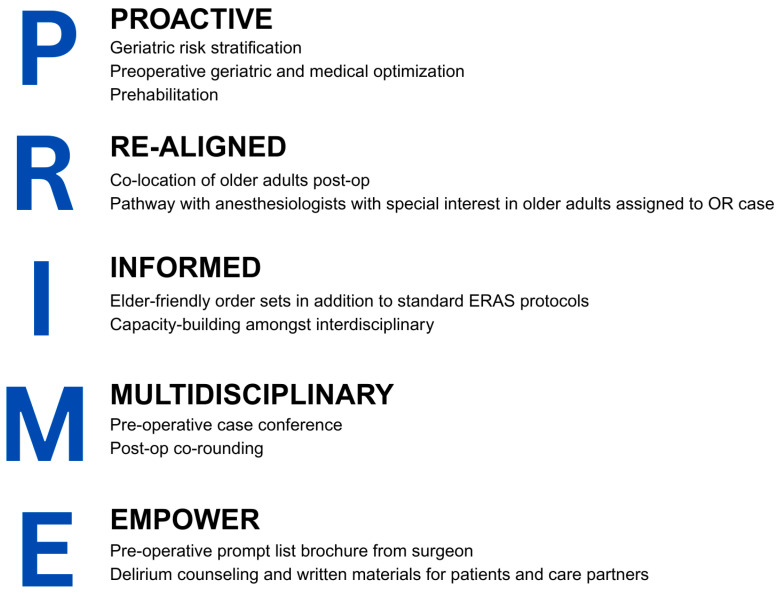
Principles of the PRIME whole pathway perioperative geriatrics strategy. OR = operating room, ERAS = Enhanced Recovery After Surgery.

The Older Adults Surgery and Oncology Program at St. Michael’s Hospital established a whole pathway perioperative geriatrics strategy, PRIME (see Figure 1). The strategy integrates geriatric principles and embeds clinicians with geriatrics expertise into the care pathway of older adults undergoing surgery from preoperative through to postoperative care. All patients aged 70 years and older received the PRIME strategy. The details of the care pathway are provided below in accordance with the Template for Intervention Description and Replication (TIDieR) guidelines [16].

Patients are initially assessed by the surgeon, who outlines the surgical plan, including the risks, benefits, and alternatives of the proposed procedure, as well as a tentative operative date. The surgeon may also identify any geriatric syndromes of concern. An electronic referral is then sent to the geriatrician for a preoperative assessment, with a target wait time of less than three weeks. The patient is then seen in-person in the geriatric clinic for a CGA conducted by the geriatrician and a nurse, with a trained interpreter present if needed. The clinic assessment includes evaluation of comorbidities, frailty (using the Rockwood Clinical Frailty Scale [17]), medications, and geriatric syndromes (including mood, vision, hearing, pain, nutrition, falls, continence, and sleep). Cognitive screening is performed using the Mini-Cog [18] or another validated tool including Montreal Cognitive Assessment, Mini Mental State Examination, or Rowland Universal Dementia Assessment Scale, based on clinical discretion. Functional status (activities and instrumental activities of daily living) and social history are also assessed. CGA recommendations may include preoperative investigations and medication adjustments specific to the perioperative period. Patients are counseled in person, with supplemental written materials, regarding geriatric-specific risks (e.g., delirium and functional decline) and associated mitigation strategies. Interventions are recommended to optimize preoperative status, such as medical management, engaging in regular physical activity for prehabilitation, increased protein intake, substance use cessation, and addressing geriatric-specific risks. Patients were offered enrollment in the PREPARE prehabilitation trial [19], however participation was not tracked. Advanced care planning (identification of a substitute decision-maker, code status, and discussion of values and preferences) is documented. Following the CGA, the geriatrician sends a summary email to the surgeon and the surgical allied health team outlining specific postoperative interventions tailored to the patient. Patients are subsequently assessed by an anesthesiologist within one week prior to surgery. If the case is complex, further coordination may occur via telephone or asynchronous email among the surgical, geriatric, and anesthesia teams to develop an optimal surgical, anesthetic, and recovery plan. If a patient is admitted for urgent surgery, they receive a CGA from the inpatient geriatric consultant, with a target time of within 24 h of admission. Postoperative care is provided by the surgical team, including the surgeon and allied health professionals (nursing, occupational therapy, social work, physiotherapy, dietitian, and case manager), with co-management by a geriatrician and a clinical nurse specialist in geriatrics immediately postoperatively, and subsequently on an as-needed basis. The clinical nurse specialist also supports the surgical allied health team by providing education on the management of geriatric syndromes. Co-rounding between the surgeon and geriatrician is arranged as needed. All patients undergoing elective surgery follow the standard Enhanced Recovery After Surgery (ERAS) protocol [20]. After discharge, a summary of the hospitalization is sent to the primary care provider, and all patients receive follow-up from the surgeon.

### 2.3. Data Collection

Data were collected by chart review using a chart abstraction guide with standardized definitions for variables and outcomes (see Supplementary Table S1). Chart reviews were conducted by three researchers (GJ, RF, CW). Uncertainties were resolved by discussion between researchers. Baseline characteristics collected from the CGA included comorbidities, medications, substance use, functional status, mobility aid use, housing type, fall history, dementia or cognitive impairment, and Clinical Frailty Scale. American Society of Anesthesiologists Physical Status classification system (ASA class), height, and weight were taken from the intraoperative anesthesia or nursing records. If no ASA class was recorded, then ASA class was determined based on known comorbidities by one researcher. Cancer or pre-cancer type and surgical procedure were obtained from the surgical and operating room notes.

Eight structural and seven process quality indicators previously described for the evaluation of geriatric co-management models were collected (see Supplementary Table S1) [14]. Structural measures were obtained through review of program documents and policies. Process measures were abstracted through chart review.

CGA prompted actions and interventions were collected from chart review and classified into nine categories as defined from prior literature: new diagnosis or clinical findings; long-term condition medication changes; lifestyle advice; therapy interventions; perioperative shared decision making and anticipatory care planning; preoperative multispecialty discussion, preoperative referrals, and investigations; surgical admission planning including perioperative medication changes pertinent to the immediate preoperative period; advice on anticipated postoperative complications; or long-term condition management) [15]. Each recommendation, such as a specific medication change or therapy referral, was counted individually to provide the total number of recommendations per patient.

Outcome quality indicators collected included those developed through a Delphi process specifically for geriatric co-management models [14]. Other surgical (any complication, serious complication, 30-day mortality, 30-day readmission, discharge to rehabilitation or long term care, length of stay) and geriatric (delirium, functional decline, new mobility aid use, pressure ulcer) outcomes included in the American College of Surgeons National Surgical Quality Improvement Program (ACS NSQIP) Surgical Risk Calculator were collected from chart review based on the ACS NSQIP definitions (Supplementary Table S1).

### 2.4. Statistics

Data were presented as total counts and proportions, mean (standard deviation) for normally distributed data, and median (interquartile range) for non-normal data using Microsoft Excel.

### 2.5. Research Ethics

The study was approved by Research Ethics Board at St Michael’s Hospital (REB# 22-202).

## 3. Results

### 3.1. Patient Characteristics

106 patients were included for analysis (119 patient charts were identified; 13 patients were excluded due to a non-cancer diagnosis). Baseline characteristics are presented in Table 1. Mean age was 79.5 years and 62% were male. Most patients were living with at least very mild frailty. The most common reason for surgery was colorectal cancer (*n* = 84).

### 3.2. Structural Indicators

Most (5/8) structural indicators were implemented (see Table 2). Structural quality indicators missing from this care pathway included geriatric order sets, standardized education for new staff on geriatric syndromes, and prevention/management protocols for some geriatric syndromes.

### 3.3. Process Indicators

In terms of process indicators (see Table 2), co-management started preoperatively or within 24 h of hospital admission in 96.2% (*n* = 102). 6.6% (*n* = 7) received CGA during the hospitalization as these were non-elective cases. There was high level of adherence to screening for geriatric syndromes (see Table 2). Structured interdisciplinary meetings have not yet been established.

### 3.4. CGA Prompted Actions and Interventions

All patients received at least one CGA prompted action or intervention, with a median number of 17 (IQR 14–20).

(1) New diagnoses or clinical findings

56 patients (52.8%) received a total of 83 new diagnoses. The most common were falls (*n* = 17, 16.0%), mild cognitive impairment (MCI) or dementia (*n* = 14, 13.2%), low B12 (*n* = 11, 10.4%), anemia (*n* = 7, 6.6%), depression (*n* = 5, 4.7%), and iron deficiency (*n* = 3, 2.8%).

(2) Long-term condition medication changes

54 patients (48.1%) had a recommendation for at least one long-term condition medication change for a total of 93 recommended medication changes, not including medication changes specific to the perioperative period. Of the long-term condition medication changes recommended, nine were for tapering psychotropic medications, seven were for discontinuing potentially inappropriate medications, and eight were for changing to a safer alternative medication. Six patients were recommended to start an antiresorptive medication for osteoporosis. The most common medications initiated included vitamin B12 supplementation (*n* = 19, 17.9%), vitamin D supplementation (*n* = 8, 7.6%), aspirin for secondary prevention (*n* = 8, 7.6%), and thiamine for patients using alcohol daily (*n* = 8, 7.6%). At the time of discharge, 56 (60.2%) of the medication changes were implemented.

(3) Lifestyle advice

102 patients (96.2%) received lifestyle advice, including nutritional recommendations specific to the perioperative period (*n* = 98, 92.5%), cessation of alcohol in the week leading up to surgery (*n* = 76, 71.7%), and smoking cessation (*n* = 9, 8.5%).

(4) Therapy interventions

98 patients (92.5%) received preoperative therapy interventions, including instructions on prehabilitation exercise (*n* = 89, 84.0%), referral for an occupational therapy home safety assessment (*n* = 8, 7.6%), referral to physiotherapy (*n* = 4, 3.8%), and information on cognitive prehabilitation (*n* = 2, 1.9%). Referrals were also made to the Alzheimer’s Society (*n* = 7, 6.6%) and the Learning the Ropes program for mild cognitive impairment (*n* = 4, 3.8%) [21].

(5) Perioperative shared decision making

There were ten patients, excluded from the study analysis, who did not proceed with surgery following the CGA and multidisciplinary team discussion. Five patients opted for non-operative management due to severe functional decline (*n* = 2), complex medical comorbidities (*n* = 2) or surgery not in line with their preferences (*n* = 1). Four patients opted for palliative care after a recommendation for supportive management and/or symptom control with radiation. One patient chose not to pursue surgery after further investigations.

(6) Preoperative investigations or referrals

90 patients (84.9%) were recommended a total of 171 preoperative investigations or referrals. The most common investigations were B-type natriuretic peptide (BNP) for cardiac risk stratification (*n* = 74, 69.8%), ferritin (*n* = 13, 12.3%), hemoglobin A1C (*n* = 11, 10.4%), complete blood count (*n* = 11, 10.4%), electrocardiogram (*n* = 7, 6.6%), and echocardiogram (*n* = 6, 6.6%). The most common referrals were to hematology (*n* = 6, 6.6%), endocrinology (*n* = 5, 4.7%), and cardiology (*n* = 2, 1.9%). At the time of surgery, 141/171 (82.5%) of the recommended preoperative investigations and referrals were completed.

(7) Surgical admission planning including perioperative medication management

105 patients (99.1%) had medication changes recommended for the perioperative period. The most common were antihypertensives (*n* = 44, 41.5%), anticoagulation (*n* = 24, 22.6%), diabetes medications (*n* = 30, 28.3%), and diuretics (*n* = 12, 11.3%). The surgeon received recommendations for medications including venous thromboembolism prophylaxis (*n* = 72, 67.9%), scheduled acetaminophen for pain (*n* = 53, 50.0%), thiamine supplementation (*n* = 30, 28.3%), salbutamol/ipratropium as needed (*n* = 7, 6.6%), and intravenous iron (*n* = 11, 10.4%). There were a total of 363 medication recommendations specific to the perioperative period. 340 (93.7%) of the perioperative medication recommendations were implemented. 82 patients (77.4%) received a personalized admission plan with 135 specific recommendations including visitation flexibility (*n* = 64, 60.4%), provision of a bed by the window (*n* = 12, 11.3%), and proactive social work referral (*n* = 5, 4.7%).

(8) Advice on anticipated postoperative complications

105 patients (99.1%) received information on potential perioperative complications. The most common complications discussed were cardiac (*n* = 104, 9.8%), delirium (*n* = 101, 95.3%), respiratory (*n* = 55, 51.9%), functional decline (*n* = 45, 42.5%), urinary retention (*n* = 23, 21.7%), and nutrition (*n* = 21, 19.8%).

(9) Long-term condition management

74 patients (69.8%) had recommendations made for management of long-term conditions, either to the primary care provider or referral to a specialist. The most common recommendations were for bone mineral density testing (*n* = 23, 21.7%) in falls risk, cognitive reassessment (*n* = 14, 13.2%), and hearing assessment (*n* = 6, 5.7%). 14 patients (13.2%) had referrals made to other specialists.

### 3.5. Outcome Indicators

Observed proportions of outcome quality indicators are outlined in Table 3. Approximately one quarter of patients experienced a surgical complication (25.5%) and one fifth experienced delirium (21.7%). At the time of discharge 21 patients (19.8%) met criteria for functional decline (see Supplementary Table S1).

## 4. Discussion

This retrospective chart review of a collaborative geriatrics and general surgery care pathway demonstrated high adherence to most structural and process quality indicators and led to many tailored CGA interventions. Nearly all patients (96.2%) received a CGA preoperatively or within 24 h of hospital admission. The CGA identified 83 new diagnoses, and over half of patients had a recommended long-term medication change. Geriatric specific considerations for surgery were addressed, with high adherence to delirium counseling (95.3%) and implementation of perioperative medication recommendations (94%). The most common complications observed were surgical complications (25.5%), delirium (21.7%), and functional decline (19.8%).

Studies of geriatric co-management or geriatric assessment in older adults undergoing gastrointestinal cancer surgery have reported variable impacts on complications, delirium, readmission, and length of stay—likely reflecting the heterogeneity of complex interventions [22,23,24]. Detailed reporting of intervention components, including process evaluations assessing adherence to the intervention, is essential to support the adoption of effective care models.

Donabedian’s framework suggests that structural measures influence process measures, which in turn affect outcomes [25]. The presence of most structural indicators in our collaborative care model may have enabled the high performance across the process indicators. In particular, the enrollment of all patients over age 70 is a significant strength, as well as the regular availability of the geriatrics service during the inpatient hospital stay. However, some structural components were lacking. Standardized order sets were deferred due to the organization’s planned transition to a new electronic medical record system. Although informal education was provided ad hoc, systematic and structured education on geriatric syndromes was not integrated into the care model at the time of its establishment. These represent areas for future program improvement.

Compared to the Perioperative Medicine for Older People undergoing Surgery (POPS) service in the United Kingdom, our CGA led to a higher number of actions and interventions, exceeding their reported median of 9 (IQR 6–12) [15]. This may reflect country-specific differences in practice scope and patterns.

While implementation of perioperative medication recommendations was high, adherence was lower for preoperative investigations, referrals, and changes to long-term condition medications. Some patients may have had these investigations conducted outside of the hospital and thus were not captured on chart review. Additionally, since the chart review only had data at the time of discharge, recommended medication changes intended for primary care follow-up after recovery from surgery were not captured.

Our observed surgical complication rate was lower than previously reported in the literature. In a meta-analysis of colorectal cancer patients, the rate of any complication was 46.9% in open and 34.6% in the laparoscopic procedures [26], both higher than our observed complication rate. The delirium rate in our study was comparable to the median incidence of delirium as reported in a meta-analysis of older adults undergoing gastrointestinal cancer surgery [27]. Delirium is often underdiagnosed [28], however in our study, all patients were assessed postoperatively by a geriatrician, which may have improved case finding. Additionally, a substantial proportion of our cohort had baseline cognitive impairment (dementia or mild cognitive impairment), increasing their risk for post-operative delirium [29]. Functional decline and length of stay were very similar to a large retrospective study of older adults undergoing colorectal cancer surgery (19.8% vs. 20.5%, median length of stay 5 vs. 5 days) [30]. Discharge to rehabilitation or other care facilities was slightly higher than the 8.4% reported in one study [31] and lower than the 27–35% reported in others [24,32]. The differences between the studies may reflect the varied levels of frailty, comorbidity, and mixture of surgical subgroups amongst studies.

Our study has several strengths. Firstly, we assessed a large number of structural and process indicators to ensure fidelity with key components of geriatric collaborative care models. We also assessed specific CGA components and reported on adherence to recommendations, where possible. Lastly, we reported key outcome measures compared to the published literature.

Several limitations must be acknowledged. As a retrospective chart review, our findings are limited by the availability and completeness of documentation. Content from other modes of communication used by strong collaborative team models such as phone and in-person discussion, email, and secure messaging was not captured. While our study included a heterogenous group of tumor types, limiting interpretation for specific tumor subgroups, perioperative geriatric co-management models typically encompass a broad range of surgical indications [15]. The implementation of the PRIME strategy coincided with the turnover of several surgeons, limiting the feasibility of a pre-post analysis.

With an understanding of the gaps in the structural, process, and outcome quality indicators, next steps include using an evidence-based and theory-informed implementation science approach to analyze barriers and facilitators to these care gaps.

## 5. Conclusions

Implementing a comprehensive, whole-pathway perioperative geriatrics strategy is feasible and can achieve high adherence to most structural and process indicators established for collaborative care models. Comprehensive geriatric assessment led to a number of actions and interventions. Outcome quality indicators suggest favorable performance compared to existing literature. Further standardization of processes may enhance program delivery.

## Figures and Tables

**Table 1:** Baseline Characteristics.

	*n* = 106, (%)
Age (mean, standard deviation)	79.5 +/− 6.8
Male gender	62 (58.5)
Surgical Indication	
Colorectal cancer	84 (79.3)
Gastric cancer	10 (9.4)
GIST	3 (2.8)
Small bowel cancer	2 (1.9)
Pre-cancerous lesion *	7 (6.6)
Surgical Procedure	
Laparoscopic right or left hemicolectomy	50 (47.1)
Laparoscopic anterior resection (+/− diverting ileostomy)	20 (18.9)
Other laparoscopic procedure	22 (20.8)
Open laparotomy	17 (16.0)
Transanal minimally invasive surgery	3 (2.8)
Emergency Case	5 (4.7)
Disseminated cancer	12 (11.3)
Body Mass Index (mean, standard deviation)	26.7 +/− 5.0
ASA Class	
1	0
2	9 (8.5)
3	53 (50.0)
4	44 (41.5)
5	0
Clinical Frailty Scale (*n* = 105)	
1-very fit	2 (1.9)
2-well	3 (2.8)
3-managing well	11 (10.4)
4-very mild frailty	54 (50.9)
5-mildly frail	26 (24.5)
6-moderately frail	9 (8.5)
Medical History	
Hypertension on medication	72 (67.9)
Diabetes on oral medication	30 (28.3)
Diabetes on insulin	6 (5.7)
Congestive heart failure (CHF) **	4 (3.8)
Smoking status within past 1 year	9 (8.5)
Severe Chronic Obstructive Pulmonary Disorder (COPD) ***	5 (4.7)
Dialysis	0
Steroid Use	3 (2.8)
Functional Status	
Independent for basic activities of daily living	96 (90.6)
Partially dependent for basic activities of daily living	9 (8.5)
Totally dependent for basic activities of daily living	1 (0.9)
Geriatric Variables	
Fall in past 1 year	34 (32.0)
Cognitive impairment (dementia or mild cognitive impairment)	20 (18.9)
Mobility aid use	35 (33)
Surrogate signed consent	7 (6.6)

* high grade dysplasia, tubulovillous adenoma, polyps. ** newly diagnosed CHF or chronic CHF with signs and symptoms within 30 days prior to surgery. *** COPD resulting in 1 of: functional disability, hospitalization for COPD, chronic bronchodilator therapy, FEV1 < 75% predicted. ASA = American Society of Anesthesiologists Physical Status classification system.

**Table 2:** Structural and Process Indicators.

Structural Indicator	Present or Not Available
A geriatrician, ward physician, nurse with geriatric expertise, ward nurses, physiotherapist, occupational therapist, social worker and case manager	**✓** *
Program review and meeting at least once yearly	**✓**
Objective criteria to select patients	**✓**
Multidisciplinary care pathway details roles and responsibilities of the staff	**✓**
Daily availability of a member of the geriatrics team	**✓**
Evidence-based protocols for the prevention and/or management of geriatric syndromes	available for some, but not all geriatric syndromes
Educational program on geriatric syndromes for new staff at least once a year	not available
Standard geriatric order sets	not available
**Process Indicator**	***n* (%)**
Preoperative geriatric co-management or within 24 h of hospital admission	102 (96.2)
Screening completed preoperatively or within 24 h of admission	
Dementia	103 (97.2)
Delirium	106 (100.0)
Functional Status	102 (96.2)
Frailty	101 (95.3)
Falls Risk	106 (100.0)
Nutrition	102 (96.2)
Medication use	102 (96.2)
Continence	102 (96.2)
Bowels	101 (95.3)
Sleep	102 (96.2)
Vision/Hearing	102 (96.2)
Pain	106 (100.0)
Pressure ulcer risk	105 (99.1)
Documentation of advanced care planning	101 (95.3)
Mean daily rounds by geriatric team (mean, standard deviation)	2.5 +/− 1.8
Documentation of discharge plan	106 (100)
Discharge summary sent to primary care provider	103 (97.2)
Collaborative interdisciplinary meetings with the primary team and a member of the geriatric team at least twice a week	not done

* “**✓**” indicates present.

**Table 3:** Outcome Indicators.

Outcome Indicator	Total *n* = 106
**Surgical Outcomes**	
Any complication *	27 (25.5%)
Serious complication **	21 (19.8%)
30 day mortality	4 (3.8%)
30 day readmission	8 (7.5%)
3 month readmission	14 (13.2%)
Discharge to rehabilitation or long-term care	14 (13.2%)
**Geriatric Outcomes**	
Delirium	23 (21.7%)
Functional decline	21 (19.8%)
New mobility aid use	17 (16.0%)
Pressure ulcer	4 (3.8%)
Restraint use	2 (1.9%)
**Length of stay (median, IQR)**	5 (3–8) days

* any complication includes: superficial incisional SSI, deep incisional SSI, organ space SSI, wound disruption, pneumonia, unplanned intubation, PE, DVT, ventilator > 48 h, progressive renal insufficiency, acute renal failure, UTI, stroke, cardiac arrest, myocardial infarction, return to the operating room, systemic sepsis. ** serious complication includes: cardiac arrest, myocardial infarction, pneumonia, progressive renal insufficiency, acute renal failure, PE, DVT, return to the operating room, deep incisional SSI, organ space SSI, systemic sepsis, unplanned intubation, UTI, wound disruption. IQR = interquartile range.

## Data Availability

The data presented in this study may be available upon request from the corresponding author and authorization is required from the Research Ethics Board in order to maintain and respect the confidentiality and privacy of this information.

## Supplementary Materials

The following supporting information can be downloaded at: https://www.mdpi.com/article/10.3390/curroncol32090494/s1. Supplementary Table S1: Chart abstraction guide of variables, indicators, and outcomes.
