# Supplementary material for: Implementation and Outcomes of a Perioperative Geriatrics Strategy, PRIME, for Older Adults Undergoing Gastrointestinal Cancer Surgery
Source: Curr Oncol. 2025 Sep 3;32(9):494. doi: 10.3390/curroncol32090494 (PMC12468265; doi:10.3390/curroncol32090494)
Supplement: Supplementary file 1 [file curroncol-32-00494-s001.zip › curroncol-3813580-supplementary.pdf]

Table S1. Chart abstraction guide of variables, indicators, and outcomes.

Surgical Risk Variable	ACS NSQIP Definition	Variable Options
Age Group	Under 65<br>65–74<br>75–84<br>85+	0 = under 65<br>1 = age 65–74<br>2 = age 75–84<br>3 = age 85+
Functional Status	The best functional status/level of self-care demonstrated by the patient within the 30 days prior surgery.<br>In terms of BADLs (dressing, eating, toileting, bathing)<br>• Independent: The patient does not require assistance from another person for any activities of daily living. This includes a person who is able to function independently with prosthetics, equipment, or devices.<br>• Partially dependent: The patient requires some assistance from another person for activities of daily living.<br>• Totally dependent: The patient requires total assistance for all activities of daily living.	0 = independent<br>1 = partially dependent<br>2 = totally dependent
Emergency Case	The principal operative procedure must be performed during the hospital admission for the diagnosis AND the surgeon and/or anesthesiologist must report the case as emergent	0 = no<br>1 = yes
ASA Class	• ASA 1: Normal healthy patient.<br>• ASA 2: Patient with mild systemic disease.<br>• ASA 3: Patient with severe systemic disease.<br>• ASA 4: Patient with severe systemic disease that is a constant threat to life.<br>• ASA 5: Moribund patient who is not expected to survive without the operation.	1 = normal healthy<br>2 = mild systemic disease<br>3 = severe systemic disease<br>4 = severe systemic disease that is a threat to life<br>5 = moribund patient who is not expected to survive without the operation
Steroid use for chronic condition	Regular administration of oral or parenteral corticosteroid medications or immunosuppressants for a chronic medical condition, within the 30 days prior to surgery, or at the time the patient is being considered as a candidate for surgery. A one-time pulse, limited short course, or a taper of less than 10 days duration would not qualify. Long-interval injections of long-acting agents would qualify.	0 = no<br>1 = yes
Ascites within 30 days prior to surgery	The presence of fluid accumulation in the peritoneal cavity noted on physical examination, abdominal ultrasound, or abdominal CT/MRI within 30 days prior to surgery. Documentation must state either active or a history of liver disease or must state secondary to malignancy.	0 = no<br>1 = yes
Systemic sepsis within 48 hours prior to surgery	Any of the following occurring within 48 hours prior to surgery:<br>• Systemic Inflammatory Response Syndrome (SIRS)<br>• Sepsis	0 = no<br>1 = SIRS<br>2 = sepsis<br>3 = septic shock

 https://doi.org/10.3390/curroncol32090494

	• Septic Shock	
Ventilator Dependent	A patient requiring ventilator-assisted respiration at any time during the 48 hours preceding surgery. This does not include the treatment of sleep apnea with CPAP.	0 = no<br>1 = yes
Disseminated Cancer	The patient has a primary cancer that has metastasized to a major organ AND meets at least one of the following:<br>• active treatment for the cancer within one year of the surgery date. If the surgical procedure is the treatment for the metastatic cancer, answer "Yes".<br>• the patient has elected not to receive treatment for the metastatic disease<br>• the patient's metastatic cancer has been deemed untreatable	0 = no<br>1 = yes
Diabetes	The individual requires daily dosages of exogenous parenteral insulin or an oral hypoglycemic agent to prevent a hyperglycemia. A patient is not included if diabetes is controlled by diet alone.	0 = no<br>1 = oral<br>2 = insulin
Hypertension requiring medication	The patient has a diagnosis of HTN in the medical record and will require antihypertensive medication(s) within 30 days prior to surgery.	0 = no<br>1 = yes
CHF in 30 days prior to surgery	Only newly diagnosed CHF within the previous 30 days or a diagnosis of chronic CHF with signs or symptoms of CHF in the 30 days prior to surgery.	0 = no<br>1 = yes
Dyspnea	The patient's dyspnea status when they were in their usual state of health, prior to the onset of the acute illness, within the 30 days prior to the time the patient is being considered a candidate for surgery.	0 = no<br>1 = with moderate exertion<br>2 = at rest
Current smoker within 1 year	The patient has smoked cigarettes in the year prior to admission for surgery. Patients who smoke cigars or pipes or use chewing tobacco are not included.	0 = no<br>1 = yes
History of Severe COPD	Chronic obstructive pulmonary disease (such as emphysema and/or chronic bronchitis) resulting in one or more of the following:<br>• Functional disability from COPD (for example, dyspnea, inability to perform ADLs)<br>• Hospitalization in the past for treatment of COPD<br>• Chronic bronchodilator therapy with oral or inhaled agents<br>• FEV1 of <75% of predicted<br>• Do not include patients whose only pulmonary disease is asthma<br>• Do not include patients with diffuse interstitial fibrosis or sarcoidosis	0 = no<br>1 = yes
Dialysis	Acute or chronic renal failure requiring treatment with peritoneal dialysis, hemodialysis, hemofiltration, hemodiafiltration, or ultrafiltration within 2 weeks prior to surgery. If a patient requires dialysis, but refuses it, the answer to this variable will be "Yes."	0 = no<br>1 = yes
Acute renal failure	A clinical condition associated with rapid decline of kidney function. The patient meets <u>one</u> of the following:	0 = no<br>1 = yes

	• Increased BUN on two measurements AND two Cr results > 3 mg/dl (>265 μmol/L)<br>• Surgeon or physician has documented Acute Renal Failure AND one of the following:<br>o Increased BUN on two measurements<br>o Two Cr results > 3 mg/dl (>265 μmol/L)	
Mobility aid use	Preoperatively, the patient uses a mobility aid (e.g., walker, cane, wheelchair, scooter, etc.), even if used intermittently.	0 = no<br>1 = yes
Origin status on admission	Differentiate whether a patient is admitted from a home or non-home location, and-if admitted from home-identify the presence of at-home support.	0 = alone at home<br>1 = supported at home (living at home with someone or receiving PSW/assistance)<br>2 = not from home
Fall History	The patient has experienced one or more falls within one year prior to the primary procedure.	0 = no<br>1 = yes
History of dementia or cognitive impairment	The patient has dementia or cognitive impairment documented by a physician or nurse at any time prior to the primary procedure.	0 = no<br>1 = yes
Hospice or palliative care on admission	The patient is on hospice (including hospice at home) or has received palliative care at the time of admission.	0 = no<br>1 = yes
Surrogate-signed consent	The patient has severe cognitive impairment-either acute or chronic-which renders him or her incapable of understanding the informed consent discussion and therefore requires a surrogate for signed consent.	0 = no, patient signed his/her own consent<br>1 = yes, consent is signed by a surrogate
**Surgical Outcomes**	**ACS NSQIP Definition**	**Variable options**
Serious complication	Cardiac arrest, myocardial infarction, pneumonia, progressive renal insufficiency, acute renal failure, PE, DVT, return to the operating room, deep incisional SSI, organ space SSI, systemic sepsis, unplanned intubation, UTI, wound disruption.	0 = no<br>1 = yes
Any complication	Superficial incisional SSI, deep incisional SSI, organ space SSI, wound disruption, pneumonia, unplanned intubation, PE, DVT, ventilator > 48 hours, progressive renal insufficiency, acute renal failure, UTI, stroke, cardiac arrest, myocardial infarction, return to the operating room, systemic sepsis.	0 = no<br>1 = yes
Pneumonia	Infection of the lungs, diagnosed using both radiologic (i.e., infiltrate, consolidation or opacity, cavitation) and clinical (e.g., fever, leukopenia/leukocytosis, culture results, patient symptoms) criteria.	0 = no<br>1 = yes
Cardiac complication	Includes cardiac arrest or myocardial infarction.<br>• Cardiac arrest: The absence of cardiac rhythm or presence of a chaotic cardiac rhythm requiring the initiation of CPR, which includes chest compressions.<br>• Myocardial infarction: ECG changes, new elevation in troponin, or physician diagnosis.	0 = no<br>1 = yes
Surgical site infection	Includes superficial incisional SSI, deep incisional SSI or organ space SSI	0 = no<br>1 = yes

	• Superficial Incisional SSI: infection that involves only skin or subcutaneous tissue of the incision. It also includes either: purulent drainage, positive culture, signs/symptoms of infection and the incision is deliberately opened by the surgeon or diagnosis by the attending physician.<br>• Deep Incisional SSI: infection that appears to be related to the operation and involves deep soft tissues (for example, fascial and muscle layers) of the incision. It also includes either: purulent drainage, spontaneous dehiscence, deliberate opening by the surgeon, abscess involving the deep incision, or diagnosis by the attending physician.<br>• Organ Space SSI: infection that involves any part of the anatomy (for example, organs or spaces), other than the incision, which was opened or manipulated during an operation. It also includes either: purulent drainage, positive culture, abscess, or diagnosis by the attending physician.	
Urinary tract infection	Bladder infection, diagnosed using a combination of clinical symptoms and laboratory confirmation (e.g., urine culture, pyuria, positive dipstick) or initiation of appropriate antimicrobial therapy.	0 = no<br>1 = yes
Venous thromboembolism / blood clot	The identification of a new thrombus within the venous system, described in studies as present in the superficial or deep venous systems but requires therapy.<br>This diagnosis is confirmed by a duplex, venogram, CT scan or other imaging modality, AND the patient requires treatment with anticoagulation therapy and/or placement of a vena cava filter or clipping of the vena cava.	0 = no<br>1 = yes
Renal failure	Includes either progressive renal insufficiency OR acute renal failure requiring dialysis.<br>• Progressive renal insufficiency: a rise in creatinine of >2 mg/dl (176.8) from preoperative value, but with no requirement for dialysis.<br>• Acute renal failure requiring dialysis: A patient who did not require dialysis preoperatively, worsening of renal dysfunction postoperatively requiring hemodialysis, peritoneal dialysis, hemofiltration, hemodiafiltration, or ultrafiltration.	0 = no<br>1 = yes
Ileus	Prolonged Postoperative NPO or NGT Use: Prolonged NPO status or NGT use for suctioning or decompression, more than 3 days postop (POD4 or later) OR reinsertion of NGT or reinstating NPO status any time POD4 or later within 30 days.	0 = no<br>1 = yes
Anastomotic leak	There was a leak of endoluminal contents through an anastomosis. This could include air, fluid, GI contents, or contrast material. The presence of an infection/abscess thought to be related to an anastomosis, even if the leak cannot be definitively	0 = no<br>1 = yes

	identified as visualized during an operation, or by contrast extravasation, would still be considered an anastomotic leak if this is indicated by the surgeon.<br>Includes leaks:<br>• Without a documented treatment intervention<br>• Treated with NPO, Antibiotics, TPN or other non-interventional, non-operative means<br>• Treated with percutaneous/radiological/endoscopic interventional means- i.e.,- percutaneous drainage with or without indwelling drain. endoscopic stent, etc.<br>• Treated with reoperation	
Return to operating room	Return to the operating room for additional surgery that was not planned at the time of the initial surgery.	0 = no<br>1 = yes
Discharge to post-acute care	Includes discharge to one of the following facilities:<br>• A skilled care facility that was not home previously (sub-acute hospital, skilled nursing home/facility, transitional care unit, long term care facility, or ventilator bed)<br>• An unskilled care facility that was not home previously (unskilled nursing home or assisted facility)<br>• Rehab (inpatient rehabilitation facility including rehabilitation distinct part units of a hospital)<br>• Separate acute care facility	0 = no<br>1 = yes
**Geriatric Outcomes**	**ACS NSQIP Definition**	**Variable options**
Postoperative delirium	Any event of postoperative delirium-or acutely altered mental status in context of the current illness-identified by documented descriptors including: mental status change, confusion, disorientation, agitation, delirium, inappropriate behavior, inattention, hallucinations, combative (e.g., pulling out lines or tubes), etc.	0 = no<br>1 = yes
Functional decline	Functional Decline is a comparison of functional health status (i.e., a measure of a patient's need for assistance in performing Activities of Daily Living) measured at discharge versus on admission. Patients who were independent on admission experienced functional decline if they were classified as partially or totally dependent upon discharge. Partially dependent patients experienced functional decline if they were classified as totally dependent upon discharge.	0 = no<br>1 = yes
New mobility aid use	Use of mobility aid (e.g., cane, walker, wheelchair, scooter, etc.) at the time of discharge that was not present on admission.	0 = no<br>1 = yes
New/worsening pressure ulcer	Development of a new pressure ulcer or progression of a present-on-admission pressure ulcer.	0 = no<br>1 = yes
**Other Geriatric Outcomes**	**Definition**	**Variable options**
3 month readmission	Admission to hospital within 3 month of discharge	0 = no<br>1 = yes
Restraint use	Use of restraints as evidenced by mandatory nursing documentation	0 = no<br>1 = yes

Discharge to long-term care	Discharge to long-term care based on hospital discharge summary	0 = no<br>1 = yes
**Process Indicators**	**Definition**	**Variable options**
Pre-operative co-management	CGA pre-operatively or within 24 hours of hospital admission based on clinic, admission, and discharge notes. Also includes patients initially admitted under another service, but who received a CGA within 24 hours of transfer to general surgery.	0 = no<br>1 = yes
Daily rounds	Number of visits by a member of the geriatrics team (resident, fellow, geriatrician, nurse specialist) based on physician daily progress notes	Number of visits
Interdisciplinary meetings between general surgery and geriatrics	Documentation of discussion of patient care between general surgery and geriatrics based on physician daily progress notes	0 = no<br>1 = yes
Screening for the following geriatric syndromes before admission or within 24 hours of admission		
Delirium	Nursing assessment for delirium with confusion assessment method within 24 hours of admission	0 = no<br>1 = yes
Dementia	Cognitive testing with mini-cog, MMSE, MOCA, or RUDAS	0 = no<br>1 = yes
Function	Baseline function, including ADLs and IADLs, documented	0 = no<br>1 = yes
Fall risk	Falls assessment by geriatrician or standardized falls risk assessment by nursing	0 = no<br>1 = yes
Social history and environment	Social history obtained by geriatrician, including living situation, place of birth, education, and employment history	0 = no<br>1 = yes
Comorbidity	Comorbidities as assessed by geriatrician	0 = no<br>1 = yes
Pressure ulcer risk	Standardized assessment with Braden scale by nursing within 24 hours of admission	0 = no<br>1 = yes
Pain	Assessment of pain by geriatrician before admission or by nurse	0 = no<br>1 = yes
Nutrition	Nutritional history obtained by geriatrician	0 = no<br>1 = yes
Continence	Continence history obtained by geriatrician	0 = no<br>1 = yes
UTI	Assessment for signs or symptoms of UTI by geriatrician	0 = no<br>1 = yes
Bowels	Bowel history obtained by geriatrician	0 = no<br>1 = yes
Hearing	History of hearing concerns obtained by geriatrician	0 = no<br>1 = yes
Vision	History of vision concerns obtained by geriatrician	0 = no<br>1 = yes
Sleep	History of sleep concerns obtained by geriatrician	0 = no<br>1 = yes
Medication use	Detailed medication review performed by a geriatrician	0 = no<br>1 = yes
Frailty	Frailty assessment using Clinical Frailty Scale by a geriatrician	0 = no<br>1 = yes
Documentation of Advanced Care Planning	Documented discussion of substitute decision maker, code status, preferences and values by a geriatrician	0 = no<br>1 = yes

Documentation of discharge plan	Discharge summary with documented discharge plan	0 = no<br>1 = yes
Discharge summary sent to primary care provider	Copies of discharge summary sent to primary care provider	0 = no<br>1 = yes
**CGA Prompted Intervention**	**Definition**	**Variable options**
New diagnosis	A condition not previously identified by the surgeon or prior available medical notes	0 = no<br>1 = yes
# of new diagnoses	Each diagnosis is counted as 1	Number of diagnoses
Chronic Medication Changes	Medication changes not related to perioperative period. Medications made to the family physician were included.	0 = no<br>1 = yes
# of chronic medication changes	Each medication change is counted as 1	Number of chronic medication changes
# of chronic medication changes completed	As determined by medication list at the time of discharge from hospital	Number of chronic medication changes / number of recommended chronic medication changes
Lifestyle Advice	Documentation of discussion on the following:<br>Alcohol abstinence<br>Smoking cessation<br>Nutrition	0 = no<br>1 = yes
# of lifestyle interventions discussed	Each lifestyle intervention is counted as 1	Number of lifestyle interventions
Therapy	Advice on exercise or referrals for structured exercise, occupational therapy, and other therapy resources.	0 = no<br>1 = yes
# of therapy interventions	Each therapy intervention is counted as 1	Number of therapy interventions
Pre-operative investigations or referrals	Preoperative investigations or referrals to specialists placed for the purpose of preoperative optimization. Investigations for chronic issues not interfering with surgical course were not counted.	0 = no<br>1 = yes
# of pre-operative investigations or referrals	Each pre-operative investigation (e.g., CBC, ferritin, ECG) is counted as 1<br>Each referral is counted as 1	Number of preoperative investigations<br>Number of referrals
# of pre-operative investigations or referrals completed	Pre-operative investigation results reported in electronic medical record or recorded in follow-up progress notes.<br>Consultation notes from referrals or documentation of recommendations from specialists.	Number of investigations completed<br>Number of referrals completed
Admission planning	Number of perioperative medication changes recommended. Each medication is counted as 1.<br>Number of specific recommendations for patient admission (e.g., window bed, visitors present). Each recommendation is counted as 1.	Number of perioperative medication changes<br>Number of recommendations for patient admission
# of perioperative medication changes completed	Perioperative medication changes	0 = no<br>1 = yes
Advice on complications	Documented discussion of possible complications.	0 = no<br>1 = yes

Number of complications discussed	Each surgical risk is counted as 1.	# of complications discussed
Long term condition management	Recommendations made to family physician for long term condition management or referrals placed to subspecialist	0 = no<br>1 = yes
# of recommendations for long term conditions	Each condition to be worked up or referral is counted as 1.	# of recommendations for long term conditions
